# Remote Delivery of Virtual Reality Patient Simulations for Nursing Education

**DOI:** 10.1093/geroni/igab046.236

**Published:** 2021-12-17

**Authors:** Jaime Hannans

**Affiliations:** California State University Channel Islands, Simi Valley, California, United States

## Abstract

In the midst of rapid transfers to online teaching for experiential learning opportunities in nursing clinical labs this past spring due to the pandemic, nursing simulations with immersive virtual reality (VR) in VR headsets were deemed impossible. In partnership with Embodied Labs, nursing faculty pivoted to facilitating VR using remote learning approaches in groups. In this new VR approach nursing students engaged in active learning, critical discourse, and reflection guided by faculty delivered VR scenarios remotely with in-session debriefing during discussion pause points. Complex scenarios focused on patient or family perspectives (e.g. during end-of-life care or navigating community and healthcare needs as a LGBTQ individual). These were valuable online learning opportunities for undergraduate nursing education. Student feedback was positive, and faculty perceptions indicated using VR remote learning offers rich, engaging discussion through complex topics important to nursing clinical practice.

